# Supplementary material for: Induction of human somatostatin and parvalbumin neurons by expressing a single transcription factor LIM homeobox 6
Source: eLife. 2018 Sep 25;7:e37382. doi: 10.7554/eLife.37382 (PMC6181563; doi:10.7554/eLife.37382)
Supplement: Supplementary file 1. — Supplementary Table 1: Summary of PV and SST neurons deriverd from hPSCs. Supplementary Table 2: Antibodies used in this study. Supplementary Table 3: Primers used in this study. Supplementary Table 4: Mycoplasma contamination testing of cell lines. [file elife-37382-supp1.docx]

## Supplementary file

**Induction of human Somatostatin and Parvalbumin neurons by expressing a single transcription factor LIM Homeobox 6**

Fang Yuan^1,2^, Xin Chen^4^, Kai-Heng Fang^1^, Yuanyuan Wang^5^, Mingyan Lin^6^, Shi-Bo Xu^1^, Hai-Qin Huo^1^, Min Xu^1^, Lixiang Ma^6^ , Yuejun Chen^7^, Shuijin He^4^* and Yan Liu^1,2,3,^*

1 Institute for Stem Cell and Neural Regeneration, School of Pharmacy, Nanjing Medical University, Nanjing 211166, China.

2 State Key Laboratory of Reproductive Medicine, Nanjing Medical Unveristy, Nanjing, China

3 Institute for Stem Cell and Regeneration, Chinese Academy of Sciences, Beijing 100101, China

4 School of Life Science and Technology, ShanghaiTech University, Shanghai 201210,China.

5 Department of Neuroscience, School of basic medical science, Nanjing Medical University, Nanjing 211166, China.

6 Department of Human Anatomy and Histology, Institute of Stem Cells and Regenerative Medicine, Fudan University Shanghai Medical School, Shanghai 200032, China.

7 Institute of Neuroscience, Chinese Academy of Sciences, 320 Yue-Yang Road, Shanghai

200031, China.

*Correspondence: [yanliu@njmu.edu.cn](mailto:yanliu@njmu.edu.cn) or heshj@shanghaitech.edu.cn

**Supplementary Table1.** Summary of PV and SST neurons deriverd from hPSCs.

| **Conversion/ Differentiation** | **Patterning factors** | **Converted factors** | **Cell Sort** | **Co-culture** | **PV%** | **SST%** | **Reference** |
| --- | --- | --- | --- | --- | --- | --- | --- |
| hPSC-forebrain interneurons | SB431542, BMPRIA, Y27632, DKK1, purmorphamine | N/A | FACS-sorted | Yes | 10 | 40.6 | [18] |
| hPSC-cortical interneurons | XLSB,SHH, purmorphamine | N/A | FACS-sorted | Yes | 5% | N/A | [17] |
| hPSC-forebrain interneurons | LDN193189，SB431542，IWP2，SAG | N/A | No | No | 0.87 | 1.53 | [19] |
| hPSC-gaba interneurons | N/A | ASCL1, DLX2 ,LHX6 and miR9/9*-124 | No | Yes | <1 | 24.3 | [34] |
| hPSC-gaba interneurons | N/A | ASCL1，MYT1L，DLX2 | No | Yes | NA | 9 | [20] |
| hPSC-gaba interneurons | purmorphamine | LHX6 | No | No | 21.32 | 29.33 | This study |
| hPSC-gaba interneurons | N/A | LHX6 | No | No | 5.32 | 7.66 | This study |

*, the value of SST percentage was not discribed in this paper.

# **Supplementary Table 2.** Antibodies used in this study.

| **Antibody** | **Isotype** | **Dilution** | **Source（cat.no）** |
| --- | --- | --- | --- |
| Calbindin(CB) | Rabbit IgG | 1:2000 | Abcam(AB1778) |
| Calretinin | Rabbit IgG | 1:2000 | Epitomics（7699/3H） |
| DCX | Rabbit IgG | 1:1000 | Cell Signal Technology(4604) |
| FOXG1 | Rabbit IgG | 1:1000 | Abcam(AB18259) |
| GABA | Rabbit IgG | 1:2000 | Sigma-Aldrich(A2052) |
| GAD67 | Mouse IgG | 1:1000 | Chemicon and Millipore(MAB5406) |
| GFAP | Mouse IgG | 1:2000 | Dako(Z0334) |
| Human nuclei | Mouse IgG | 1:500 | Chemicon and Millipore(MAB1281) |
| Islet1 | Mouse IgG | 1:400 | DSHB, Iowa City, IA(40.2D6) |
| Ki67 | Rabbit IgG | 1:1000 | ZYMED（180191Z） |
| Map2 | Mouse IgG | 1:1000 | Sigma-Aldrich(M1406) |
| Meis2 | Goat IgG | 1:1000 | Santa Cruz biotechnology(SC-10599) |
| Nanog | Goat IgG | 1:1000 | R&D Systems(AF1997) |
| Nestin | Rabbit IgG | 1:1000 | Santa Cruz（SC-21247 ) |
| Nkx2.1 | Rabbit IgG | 1:500 | Santa Cruz（SC-13040) |
| Olig2 | Goat IgG | 1:500 | Santa Cruz biotechnology(SC-19969) |
| PAX6 | Mouse IgG | 1:1000 | Covance Research Products(PRB-278P) |
| PAX6 | Rabbit IgG | 1:1000 | DSHB, Iowa City, IA(PAX6) |
| Parvalbumin(PV) | Rabbit IgG | 1:5000 | Sigma-Aldrich(P3088) |
| Sox1 | Goat IgG | 1:1000 | R&D Systems(MAB3369) |
| Sox2 | Goat IgG | 1:1000 | R&D Systems(BAF2018) |
| Somatostatin and | Rat IgG | 1:500 | Chemicon and Millipore(MAB354) |
| Receptor(SST) |  |  |  |
| TH | Rabbit IgG | 1:500 | Pel-Freez(P40101) |
| β-III Tubulin | Mouse IgG | 1:2000 | Sigma-Aldrich(T8660) |
| β-III Tubulin | Rabbit IgG | 1:2000 | Covance Research Products（PRB-435P） |

# **Supplementary Table 3.** Primers used in this study.

| **Human gene** | **Forward** | **Reverse** |
| --- | --- | --- |
| *GAPDH* | TCGACAGTCAGCCGCATCTTCTTT | ACCAAATCCGTTGACTCCGACCTT |
| *LHX6(1)* | ACAGATCTACGCCAGCGACT | CATGGTGTCGTAGTGGATGC |
| *LHX6(2)* | ATCTGTCTGCTCACCGCCCT | CTTGAGCAGATATCGGTCCA |
| *LHX8* | CCAAAACCAGCAAAAAGAGC | TGGCGTGCTCTACAATTCTG |
| *ISLET1* | GTTTGAAATGTGCGGAGTGTAAT | RTTCTTGCTGAAGCCGATGC |
| *SHH* | CTCGCTGCTGGTATGCTCG | ATCGCTCGGAGTTTCTGGAGA |
| *PAX6* | ACAGATCTACGCCAGCGACT | CATGGTGTCGTAGTGGATGC |
| *NKX2.1* | CGCATCCAATCTCAAGGAAT | CAGAGTGTGCCCAGAGTGAA |
| *MEIS2* | CCAGGGGACTACGTTTCTCA | TAACATTGTGGGGCTCTGTG |
| *EMX1* | TTCAATGGGAGAGGGAGAGTGCTT | CCGTCAGCCTTTGTGAATGGTGTT |
| *MASH1* | GTCTCCCGGGGATTTTGTAT | TCTCCATCTTGGCAGAGCTT |

# **Supplementary Table 4.** Mycoplasma contamination testing of cell lines.
